# Supplementary material for: Identification of photoperiod-induced specific miRNAs in the adrenal glands of Sunite sheep (Ovis aries)
Source: Front Vet Sci. 2022 Jul 22;9:888207. doi: 10.3389/fvets.2022.888207 (PMC9354845; doi:10.3389/fvets.2022.888207)
Supplement: Supplementary file 1 [file Data_Sheet_1.ZIP › Supplementary file/miRDeep2 file.zip!/miRDeep2 file/Novel_Structure/LP42A3/Novel_34.pdf]

Provisional ID	:	NC_019484.2_25352
Score total	:	1.9
Score for star read(s)	:	-1.3
Score for read counts	:	0
Score for mfe	:	1.6
Score for randfold	:	1.6
Score for cons. seed	:	
Total read count	:	240
Mature read count	:	240
Loop read count	:	0
Star read count	:	0

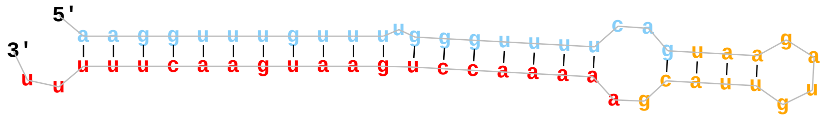

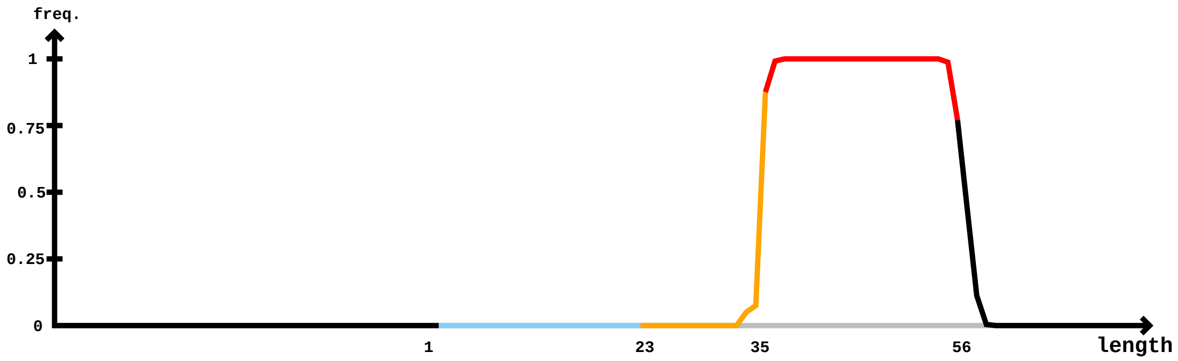

Star Mature

```
5'- acacugacugugaauauuuaaaauacuggguuggccaaaagguuuguuuugggguuuucaguaagauguuacgaaaaaccugaaugaacuuuuuguccaaccuaauauggcag  -3'   exp
    .(((.....)))..........(((.((((((((.((((((((((((((.(((((((..((((....))))..))))))))))))))))))))))).)))))))).))).....   reads   mm   sample
    .......................................................................Agaaaaaccugaaugaacuuu.....................   1       1    seq
    .......................................................................cgaaaaaccugaaugaacuuuu....................   1       0    seq
    .......................................................................Agaaaaaccugaaugaacuuuu....................   10      1    seq
    ........................................................................gaaaaaccugaaugaacuuu.....................   1       0    seq
    ........................................................................gaaaaaccugaaCgaacuuuu....................   1       1    seq
    ........................................................................gaaaaaccugaaugaacuuuu....................   2       0    seq
    ........................................................................gaaaaaUcugaaugaacuuuuu...................   1       1    seq
    ........................................................................gaaaaaccugaaugaacCuuuug..................   1       1    seq
    .........................................................................aaaaaccugaaCgaacuuu.....................   1       1    seq
    .........................................................................aaaaaccAgaaugaacuuuu....................   3       1    seq
    .........................................................................aaaaaccCgaaugaacuuuu....................   2       1    seq
    .........................................................................aaaaaUcugaaugaacuuuu....................   23      1    seq
    .........................................................................aaaaaccugaaCgaacuuuu....................   7       1    seq
    .........................................................................aaaaaccAgaaugaacuuuuu...................   2       1    seq
    .........................................................................aaaaaccugaaugGacuuuuu...................   1       1    seq
    .........................................................................aaaaaccCgaaugaacuuuuu...................   1       1    seq
    .........................................................................aaaaaccugaaugaacuuuuu...................   14      0    seq
    .........................................................................aaaaaccugaaGgaacuuuuu...................   3       1    seq
    .........................................................................aaaaaUcugaaugaacuuuuu...................   65      1    seq
    .........................................................................aaaaaccugaaugaacuuuuC...................   1       1    seq
    .........................................................................aaaaaUcugaaugaacuuuuug..................   42      1    seq
    .........................................................................Gaaaaccugaaugaacuuuuug..................   1       1    seq
    .........................................................................aaaaaccugaaugaGcuuuuug..................   1       1    seq
    .........................................................................aaaaaccugaaugaacuuuuug..................   13      0    seq
    .........................................................................aaaaaccugaaugaacuuuuugA.................   1       1    seq
    .........................................................................aaaaaccugaaugaacuuuuugC.................   3       1    seq
    .........................................................................Gaaaaccugaaugaacuuuuugu.................   1       1    seq
    .........................................................................aaaaaccugaaugaacuuuuugu.................   7       0    seq
    ..........................................................................aaaaccugaaugaacuuuu....................   2       0    seq
    ..........................................................................aaaCccugaaugaacuuuuu...................   1       1    seq
    ..........................................................................aaaaccAgaaugaacuuuuu...................   2       1    seq
    ..........................................................................Gaaaccugaaugaacuuuuug..................   1       1    seq
    ..........................................................................aaaaccCgaaugaacuuuuug..................   6       1    seq
    ..........................................................................aaCaccugaaugaacuuuuug..................   1       1    seq
```

```
                                      Star                               Mature
acacugacugugaauauuuaaaauacuggguuggccaaaagguuuguuuuggguuuucaguaagauguuacgaaaaaccugaaugaacuuuuuguccaaccuaauauggcag
.........................................................................aaaaccugaaugaacuuuuugC.................   2    1    seq
.........................................................................aaaaccugaaugaacuuuuugu.................   12   0    seq
.........................................................................aaaaccugaaugaacuuuuuguc................   1    0    seq
..........................................................................aaaccugaaugaacuuuu....................   1    0    seq
..........................................................................aaaccAgaaugaacuuuuu...................   1    1    seq
```
